# SGLT2 inhibitors, semaglutide, and finerenone in diabetic kidney disease: a Bayesian network meta-analysis

**DOI:** 10.3389/fphar.2026.1882227

**Published:** 2026-09-24

**Authors:** Zongze He, Xu Wang, Li Zhang, Xiangmei Chen

**Affiliations:** Senior Department of Nephrology, Chinese PLA General Hospital, State Key Laboratory of Kidney Diseases, National Clinical Research Center for Kidney Diseases, Beijing Key Laboratory of Medical Devices and Integrated Traditional Chinese and Western Drug Development for Severe Kidney Diseases, Beijing Key Laboratory of Digital Intelligent TCM for the Prevention and Treatment of Pan-vascular Diseases, Beijing, China

**Keywords:** Bayesian network meta-analysis, cardiorenal protection, diabetic kidney disease, finerenone, FLOW trial, GLP-1 receptor agonists, semaglutide, SGLT2 inhibitors

## Abstract

**Background:**

Despite optimal renin-angiotensin system (RAS) blockade, patients with diabetic kidney disease (DKD) face substantial residual cardiorenal risk. The 2024 FLOW trial established semaglutide as the first GLP-1 receptor agonist with a dedicated primary renal outcome benefit. We conducted a Bayesian network meta-analysis comparing SGLT2 inhibitors, semaglutide, and finerenone—the three therapies with contemporary phase 3 cardiorenal evidence—to define their comparative efficacy and safety hierarchy in type 2 diabetes with chronic kidney disease.

**Methods:**

We systematically searched PubMed, Embase, and the Cochrane Library through 31 July 2025 for phase 3 randomized trials reporting kidney, cardiovascular, or safety outcomes. We treated FIDELIO-DKD and FIGARO-DKD as separate evidence nodes to preserve between-trial heterogeneity. For DAPA-CKD and EMPA-KIDNEY, only the pre-specified type 2 diabetes subgroup data were used. Efficacy was synthesized using hazard ratios (HRs) with 95% credible intervals (CrIs); safety was modeled using a binomial likelihood for treatment discontinuation. Treatment hierarchy was quantified by the surface under the cumulative ranking curve (SUCRA). Four pre-specified sensitivity analyses evaluated robustness (±SCORED trial; fixed-vs. random-effects models).

**Results:**

Six trial-level datasets (CREDENCE, DAPA-CKD T2D subgroup, EMPA-KIDNEY T2D subgroup, FIDELIO-DKD, FIGARO-DKD, FLOW; N = 20,521) were included in the main analysis. All trials were at low risk of bias. Kidney outcomes: SGLT2 inhibitors demonstrated the greatest risk reduction (HR 0.70, 95% CrI 0.64–0.76; SUCRA 94.5%), followed by semaglutide (HR 0.76, 95% CrI 0.66–0.88; SUCRA 68.0%) and finerenone (HR 0.84, 95% CrI 0.77–0.92; SUCRA 37.5%). The indirect comparison between SGLT2 inhibitors and finerenone reached statistical significance (HR 0.83, 95% CrI 0.73–0.95; posterior probability of SGLT2i superiority >99.9% [fixed effects]; 96% [random effects]). In contrast to prior NMAs that pooled finerenone trials via the FIDELITY estimate (HR 0.77), our approach of separating FIDELIO-DKD and FIGARO-DKD yielded a pooled finerenone HR of 0.84, allowing this indirect detection. This finding is sensitive to the analytic decision to separate the two finerenone trials and should be interpreted as hypothesis-generating. Semaglutide was numerically but not statistically superior to finerenone (HR 0.90, 95% CrI 0.76–1.07; posterior probability = 88%).

**Cardiovascular outcomes:**

All three active agents significantly reduced cardiovascular risk versus placebo, with no statistically significant differences between active agents. Robustness: All key findings were stable across four sensitivity analyses; the posterior probability of SGLT2 inhibitor superiority to finerenone for kidney protection remained ≥96% in all specifications.

**Conclusion:**

SGLT2 inhibitors provide the greatest kidney protection in this network; when the two finerenone trials are analyzed separately, an indirect statistically significant SGLT2i advantage emerges (HR 0.83, 95% CrI 0.73–0.95 [fixed effects]). This finding is analytic-choice-dependent and hypothesis-generating. Semaglutide provides substantial renal protection that is numerically superior to finerenone. These findings support a four-pillar framework—RAS blockade and SGLT2 inhibition as the foundational combination, with semaglutide and finerenone as complementary phenotype-matched additions—and provide a probabilistic evidence base for individualized prescribing.

## 1 Introduction

Diabetic kidney disease remains the leading cause of end-stage kidney disease worldwide, contributing substantially to global morbidity and mortality (Bikbov et al., 2020). For several decades, inhibition of the renin-angiotensin system (RAS) provided the only evidence-based pharmacological strategy for slowing its progression. Even with optimal RAS blockade, however, patients with diabetic kidney disease continue to face substantial residual risk of disease progression, cardiovascular events, and premature mortality, driving the search for complementary therapies that target alternative mechanisms of nephropathic injury including inflammation, fibrosis, and hemodynamic dysregulation (Barrera-Chimal et al., 2022).

Three drug classes have reshaped this landscape over the past decade (de Boer et al., 2022; Navaneethan et al., 2023). Sodium-glucose cotransporter 2 (SGLT2) inhibitors established themselves as foundational kidney-protective therapy through landmark trials including CREDENCE and DAPA-CKD (Perkovic et al., 2019; Heerspink et al., 2020), with subsequent replication in non-diabetic CKD (EMPA-KIDNEY) (Herrington et al., 2023). The non-steroidal mineralocorticoid receptor antagonist finerenone demonstrated significant cardiorenal benefit in FIDELIO-DKD and FIGARO-DKD (Bakris et al., 2020; Pitt et al., 2021) (pooled in the FIDELITY analysis), targeting the inflammatory and fibrotic consequences of mineralocorticoid receptor overactivation (Agarwal et al., 2022). Most recently, the FLOW trial provided the first dedicated primary-endpoint renal outcome evidence for a glucagon-like peptide-1 receptor agonist (GLP-1 RA), with semaglutide producing a 24% reduction in the composite kidney outcome (Perkovic et al., 2024). Because currently only one agent in the ns-MRA class (finerenone) and one agent in the GLP-1 RA class (semaglutide) have phase 3 evidence for the primary renal endpoint, we refer to these therapies at the individual-drug rather than the drug-class level throughout this analysis.

Several network meta-analyses have previously compared these agents in diabetic kidney disease (Yamada et al., 2021; Apperloo et al., 2024), but all were conducted before FLOW established the first definitive renal outcome evidence for a GLP-1 RA. Prior syntheses therefore relied on kidney outcomes reported as exploratory secondary endpoints within cardiovascular outcome trials - a substantially weaker evidence base. These analyses also treated the finerenone evidence as a single pooled estimate derived from FIDELITY, which obscures substantive differences between FIDELIO-DKD and FIGARO-DKD in inclusion criteria, primary endpoints, and patient populations.

We therefore conducted a Bayesian network meta-analysis with several methodological refinements intended to yield a more faithful comparative picture. We incorporated the primary renal outcome data from FLOW; we treated FIDELIO-DKD and FIGARO-DKD as separate evidence nodes rather than pooling them; we restricted extraction to type 2 diabetes subgroups from trials (DAPA-CKD, EMPA-KIDNEY) that enrolled mixed populations; and we performed four pre-specified sensitivity analyses spanning different model specifications and the inclusion of sotagliflozin (SCORED). The objectives were (1) to quantify pairwise differences in kidney, cardiovascular, and safety outcomes among SGLT2 inhibitors, semaglutide, and finerenone; (2) to rank them probabilistically across these three outcome domains; and (3) to evaluate the robustness of these estimates to model choices. Our findings are intended to inform individualized prescribing within the four-pillar framework—RAS blockade, SGLT2 inhibition, semaglutide, and finerenone—that now defines contemporary diabetic kidney disease pharmacotherapy.

## 2 Methods

### 2.1 Study protocol and guidelines

This systematic review and network meta-analysis (NMA) was conducted and reported in accordance with the Preferred Reporting Items for Systematic Reviews and Meta-Analyses extension for Network Meta-Analyses (PRISMA-NMA) guidelines (Hutton et al., 2015). The methodological framework followed the Cochrane Handbook for Systematic Reviews of Interventions (Higgins et al., 2019). The review protocol was not prospectively registered in PROSPERO or a comparable registry; all pre-specifications described below were documented internally prior to commencement of data extraction and analysis.

### 2.2 Search strategy and selection criteria

We systematically searched PubMed, Embase, and the Cochrane Central Register of Controlled Trials from database inception through 31 July 2025. The search strategy combined controlled vocabulary (MeSH/Emtree) and free-text terms across three conceptual axes: (1) intervention — “SGLT2 inhibitor,” “dapagliflozin,” “empagliflozin,” “canagliflozin,” “sotagliflozin,” “GLP-1 receptor agonist,” “semaglutide,” “liraglutide,” “dulaglutide,” “non-steroidal mineralocorticoid receptor antagonist,” “finerenone”; (2) population — “type 2 diabetes,” “diabetic kidney disease,” “diabetic nephropathy,” “chronic kidney disease”; (3) study design — “randomized controlled trial,” “phase 3.” No language or date restrictions were applied. The full search strategy is provided in Supplementary Table S1. Two reviewers (Z.H. and X.W.) independently screened titles and abstracts, followed by full-text review of potentially eligible articles. Discrepancies were resolved by consensus or adjudication by a third reviewer (L.Z.).

### 2.3 Eligibility criteria

Trials were eligible if they met the following pre-specified PICOS criteria. Population: Adults with type 2 diabetes and chronic kidney disease, defined by reduced eGFR and/or elevated UACR per individual trial thresholds. For trials enrolling both diabetic and non-diabetic populations, T2D-specific data were used where available with full precision. For DAPA-CKD, the pre-specified T2D subgroup analysis was used; for EMPA-KIDNEY, the overall trial HR was applied (see §2.4). Interventions: (1) a selective SGLT2 inhibitor (dapagliflozin, empagliflozin, or canagliflozin); (2) semaglutide; or (3) finerenone. Comparator: Placebo added to standard background therapy including RAS inhibition. Outcomes: At least one of the following: composite kidney outcome, composite cardiovascular outcome, or treatment discontinuation. Study design: Phase 3 randomized controlled trial with follow-up duration ≥12 months. “Large-scale” was operationalized as a sample size ≥2,000 participants randomized.

### 2.4 Pre-specified study-level decisions

Three *a priori* decisions warrant explicit documentation. (1) Separation of FIDELIO-DKD and FIGARO-DKD: Although the FIDELITY pooled analysis provides the most powerful estimate of finerenone’s effect, it combines two trials that differed substantively in inclusion criteria, primary endpoints, and follow-up durations. To preserve between-trial heterogeneity information, we treated FIDELIO-DKD and FIGARO-DKD as separate nodes of evidence in all analyses. (2) Handling of DAPA-CKD and EMPA-KIDNEY: Both trials enrolled mixed populations. For DAPA-CKD, we used the pre-specified T2D subgroup analysis (Wheeler et al., Lancet DE 2021; N = 2,906; HR 0.64, 95% CI 0.52–0.79). For EMPA-KIDNEY, the primary publication does not report the T2D subgroup kidney composite HR with full precision (95% CI and event counts); we therefore used the overall trial HR (0.72, 95% CI 0.64–0.82), supported by a non-significant diabetes-vs-non-diabetes interaction (p = 0.62). A sensitivity analysis excluding EMPA-KIDNEY entirely (Sensitivity E) was pre-specified. (3) Handling of SCORED: SCORED evaluated sotagliflozin, a dual SGLT1/SGLT2 inhibitor (Sridhar et al., 2024); was terminated prematurely with insufficient renal events; and revised its primary endpoint mid-trial. SCORED was therefore excluded from the main analysis but included in a pre-specified sensitivity analysis.

### 2.5 Outcome definitions

The primary outcome was the composite kidney endpoint. Because the precise components differ between trials (Supplementary Table S2), we used the trial-defined composite kidney endpoint that was either the primary outcome or the leading pre-specified kidney secondary outcome. Core components included end-stage kidney disease (ESKD), sustained eGFR decline (≥40% in CREDENCE, FIDELIO-DKD, FIGARO-DKD, EMPA-KIDNEY, FLOW; ≥50% in DAPA-CKD), and renal death (Heerspink et al., 2023). Secondary outcomes were the composite cardiovascular endpoint and permanent treatment discontinuation due to any adverse event.

### 2.6 Data extraction and risk-of-bias assessment

Two reviewers (Z.H. and X.W.) independently extracted data using a standardized form. Discrepancies were resolved by consensus. Risk of bias was assessed using the Cochrane Risk of Bias 2.0 (RoB 2.0) tool (Sterne et al., 2019), covering five domains: randomization process, deviations from intended interventions, missing outcome data, measurement of the outcome, and selection of the reported result.

### 2.7 Statistical analysis

We performed a Bayesian network meta-analysis under the consistency assumption (Dias et al., 2013). For hazard ratio outcomes (kidney and cardiovascular composites), a contrast-level normal likelihood with identity link was applied to study-specific log-hazard ratios. For treatment discontinuation, an arm-level binomial likelihood with logit link was fitted. Both fixed-effects and random-effects models were fitted, with fixed-effects estimates presented as primary given the small number of trials per node and the observed low between-study heterogeneity (τ < 0.1 across all outcomes).

Markov Chain Monte Carlo sampling was performed using the No-U-Turn Sampler implemented in PyMC (version 5.28) (Salvatier et al., 2016), with four chains of 5,000 draws after 2,000 tuning iterations per chain. Treatment hierarchy was summarized using SUCRA (Salanti et al., 2011). Pairwise comparisons were additionally characterized by the posterior probability that one treatment was superior to the comparator (P[HR < 1.0]). Given the star-shaped network topology with no closed loops, formal loop-specific inconsistency testing was not applicable (Dias et al., 2010).

### 2.8 Sensitivity analyses

Four pre-specified sensitivity analyses were performed: (1) main analysis — six trials, fixed-effects model; (2) sensitivity A — six trials, random-effects model; (3) sensitivity B — seven trials (adding SCORED), fixed-effects model; (4) sensitivity C — seven trials, random-effects model.

## 3 Results

### 3.1 Study selection and characteristics

The systematic search identified 3,452 records. After duplicate removal, title/abstract screening, and full-text review, six distinct trial-level datasets were included in the main analysis: CREDENCE, DAPA-CKD (T2D subgroup), EMPA-KIDNEY (T2D subgroup), FIDELIO-DKD, FIGARO-DKD, and FLOW. The updated PRISMA 2020 flow diagram is shown in Figure 1. The SCORED trial was excluded from the main analysis but incorporated in a pre-specified sensitivity analysis. A total of 20,521 participants with type 2 diabetes and CKD contributed to the main analysis. Baseline characteristics are presented in Table 1, grouped by therapeutic class.

**FIGURE 1:**
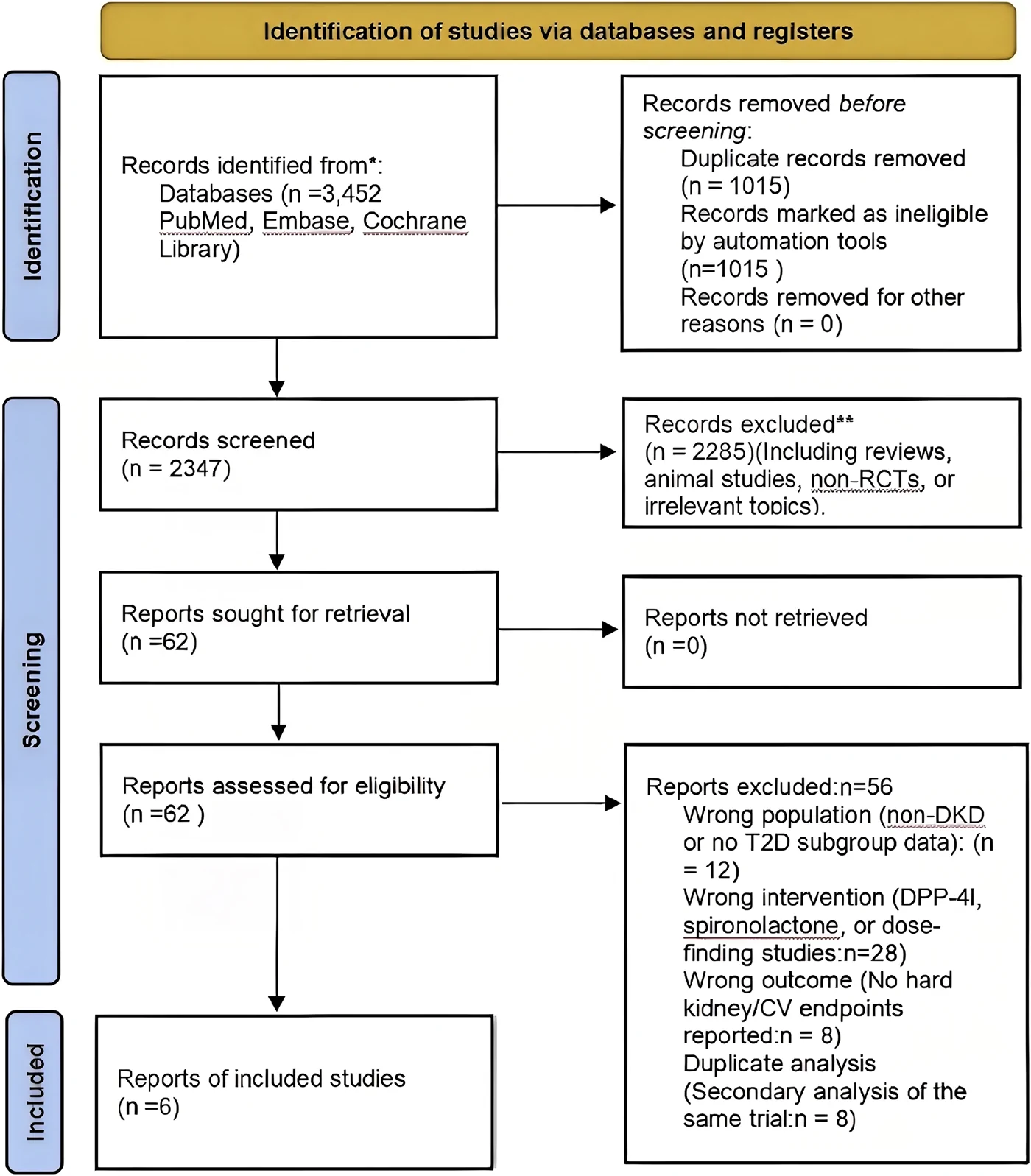
PRISMA 2020 flow diagram of study selection.

**TABLE 1:** Baseline characteristics of included trials.

Study	Year	Intervention	N (T2D)	Age (yrs)	eGFR (mL/min/1.73 m^2^)	UACR (mg/g)	ASCVD (%)	Follow-up (yrs)
*a. Selective SGLT2 inhibitor trials*								
CREDENCE	2019	Canagliflozin 100 mg vs. placebo	4,401	63.0 ± 9.2	56.2 ± 18.2	927 (463–1,833)	50.4	2.6
DAPA-CKD (T2D)	2020	Dapagliflozin 10 mg vs. placebo	2,906	64.5 ± 10.1	43.1 ± 12.4	1,019 (475–2,106)	45.5	2.4
EMPA-KIDNEY (T2D)	2023	Empagliflozin 10 mg vs. placebo	3,040	64.2 ± 10.8	36.9 ± 14.2	395 (88–1,254)	33.7	2.0
*b. Non-steroidal mineralocorticoid receptor antagonist trials*								
FIDELIO-DKD	2020	Finerenone 10/20 mg vs. placebo	5,734	65.6 ± 8.9	44.3 ± 12.6	852 (446–1,634)	45.9	2.6
FIGARO-DKD	2021	Finerenone 10/20 mg vs. placebo	7,437	64.1 ± 9.8	67.8 ± 21.7	308 (108–740)	45.8	3.4
*c. GLP-1 receptor agonist trial*								
FLOW	2024	Semaglutide 1.0 mg s.c. weekly vs. placebo	3,533	66.6 ± 9.0	47.0 ± 15.2	568 (230–1,297)	67.5	3.4

For DAPA-CKD, and EMPA-KIDNEY, baseline data reflect the pre-specified type 2 diabetes subgroup. UACR, values are medians with interquartile ranges. Background renin-angiotensin system inhibitor use exceeded 94% in all trials. Abbreviations: ASCVD, atherosclerotic cardiovascular disease; eGFR, estimated glomerular filtration rate; T2D, type 2 diabetes; UACR, urinary albumin-to-creatinine ratio.

### 3.2 Risk of bias assessment

All six included trials were judged to be at low risk of bias across all five RoB 2.0 domains, consistent with their design as large, well-conducted, blinded phase 3 randomized controlled trials. The traffic-light summary is shown in Supplementary Figure S1.

### 3.3 Network geometry

The evidence network comprised four nodes (placebo, SGLT2 inhibitor, semaglutide, and finerenone) connected by six trial-level edges anchored on the common placebo comparator (Figure 2). The SGLT2 inhibitor node was supported by three trials; the finerenone node by two trials; and the semaglutide node by one trial. No direct head-to-head randomized comparisons between active treatments were identified. Given the star-shaped topology with no closed loops, formal loop-specific inconsistency testing was not applicable; we instead evaluated transitivity by comparing effect-modifier distributions across trial placebo arms, which supported the plausibility of the transitivity assumption.

**FIGURE 2:**
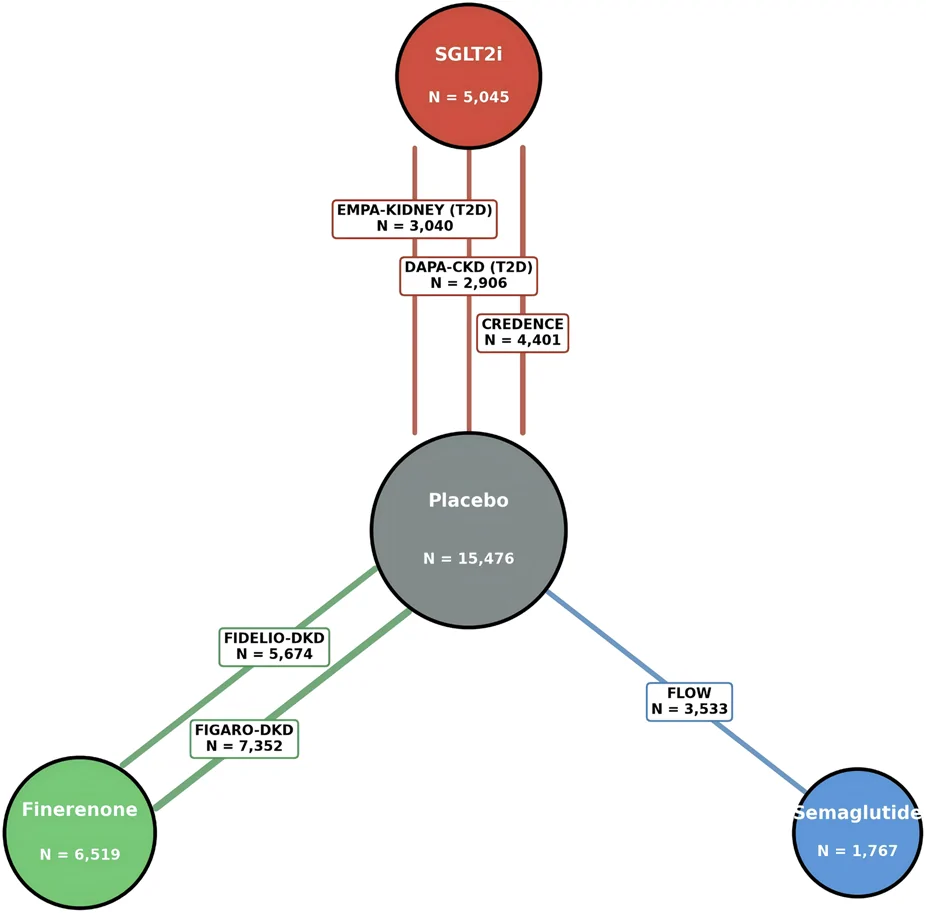
Evidence network for the Bayesian network meta-analysis. Nodes represent treatment classes; edges represent randomized controlled trials. Node size reflects total participants per treatment arm; edge thickness reflects trial sample size. The star-shaped topology (no closed loops among active treatments) means that all pairwise comparisons between active agents are derived from indirect evidence via the shared placebo comparator.

### 3.4 Primary outcome: composite kidney endpoint

All three active treatments significantly reduced the risk of the composite kidney outcome relative to placebo (Table 2). In the main analysis (fixed effects), SGLT2 inhibition was associated with the greatest risk reduction (HR 0.70, 95% CrI 0.64–0.76; random effects: HR 0.70, 95% CrI 0.59–0.81), followed by semaglutide (FE: HR 0.76, 95% CrI 0.66–0.88; RE: HR 0.76, 95% CrI 0.59–0.99) and finerenone (FE: HR 0.84, 95% CrI 0.77–0.92; RE: HR 0.84, 95% CrI 0.71–1.03).

**TABLE 2:** League table for composite kidney outcome.

​Comparator	Finerenone	Semaglutide	SGLT2i
Placebo	0.84 (0.77–0.92)*	0.76 (0.66–0.88)*	0.70 (0.64–0.76)*
Finerenone	—	0.90 (0.76–1.07)	0.83 (0.73–0.95)*
Semaglutide	—	—	0.92 (0.78–1.09)

Hazard ratios (95% credible intervals) for column treatment vs. row treatment. Values <1.00 favor the column treatment. Asterisks (*) indicate statistical significance (95% CrI excludes 1.00).

The indirect head-to-head comparison between SGLT2 inhibitors and finerenone yielded a hazard ratio of 0.83 (95% CrI 0.73–0.95), indicating a statistically significant kidney-protective advantage of SGLT2 inhibitors over finerenone. The posterior probability that SGLT2 inhibitors provided superior renal protection to finerenone was >99.9% (fixed effects) and 96% (random effects). The semaglutide-versus-finerenone comparison yielded HR 0.90 (95% CrI 0.76–1.07), numerically favoring semaglutide with a posterior probability of 88% for superiority. Semaglutide and SGLT2 inhibitors were not significantly different (HR 0.92, 95% CrI 0.78–1.09).

These NMA estimates are consistent with the individual trial results; the novel contribution of this analysis is the between-agent indirect comparisons-particularly the SGLT2i-versus-finerenone kidney comparison—which are not obtainable from any single trial and emerge only through evidence synthesis across the network.

The separation of FIDELIO-DKD and FIGARO-DKD materially changed the indirect comparison between SGLT2 inhibitors and finerenone for the kidney outcome. In prior network meta-analyses that pooled FIDELIO-DKD and FIGARO-DKD via the FIDELITY combined hazard ratio of 0.77, this comparison was not statistically significant. Preserving the two trials as separate evidence nodes shifted the pooled finerenone kidney hazard ratio to 0.84 and allowed indirect detection of the SGLT2i-versus-finerenone contrast. This finding is sensitive to the analytic decision to separate the two finerenone trials and should be considered hypothesis-generating rather than definitive.

### 3.5 Secondary outcome: composite cardiovascular endpoint

All three active agents significantly reduced the cardiovascular composite outcome relative to placebo (Table 3): SGLT2 inhibitors HR 0.79 (95% CrI 0.70–0.90), semaglutide HR 0.82 (95% CrI 0.68–0.99), and finerenone HR 0.87 (95% CrI 0.79–0.95). No statistically significant differences were observed among the three active agents in head-to-head indirect comparisons.

**TABLE 3:** League table for composite cardiovascular outcome.

​Comparator	Finerenone	Semaglutide	SGLT2i
Placebo	0.87 (0.79–0.95)*	0.82 (0.68–0.99)*	0.79 (0.70–0.90)*
Finerenone	—	0.95 (0.76–1.18)	0.91 (0.78–1.07)
Semaglutide	—	—	0.96 (0.77–1.21)

Hazard ratios (95% credible intervals) for column treatment vs. row treatment. Values <1.00 favor the column treatment. Asterisks (*) indicate statistical significance.

### 3.6 Safety: agent-specific adverse event profiles

Agent-specific adverse event profiles are the clinically meaningful guide to safety-based prescribing in this population. Gastrointestinal adverse events (nausea, vomiting, diarrhea) are the predominant driver of semaglutide discontinuation, with permanent discontinuation reported in 13.2% of FLOW participants versus 11.9% on placebo. Hyperkalemia is the characteristic safety concern with finerenone, with hyperkalemia-related discontinuation reported as 2.3% versus 0.9% (FIDELIO-DKD) and 1.2% versus 0.4% (FIGARO-DKD). SGLT2 inhibitors are associated with genitourinary infections and a small risk of diabetic ketoacidosis, which were rare causes of permanent discontinuation in the included trials. These qualitatively distinct profiles are not captured in a pooled all-cause discontinuation odds ratio and should not be compared via a single composite metric. All-cause discontinuation NMA results are reported in Supplementary Table S5 for completeness.

### 3.7 Treatment hierarchy (SUCRA)

The SUCRA analysis (Table 4) confirmed a clear treatment hierarchy. A two-dimensional SUCRA plot is presented in Figure 3.

**TABLE 4:** SUCRA values and treatment hierarchy.

Rank	Intervention	SUCRA (%)	Interpretation
*A. Composite kidney outcome*			
1	SGLT2 inhibitor	94.5	Most robust renal protection; significant advantage over finerenone
2	Semaglutide	68.0	Effective; numerically superior to finerenone
3	Finerenone	37.5	Effective vs. placebo but inferior to SGLT2i
4	Placebo	0.0	Background risk on RAS blockade
*B. Composite cardiovascular outcome*			
1	SGLT2 inhibitor	83.5	Numerically superior CV protection
2	Semaglutide	67.9	Comparable CV efficacy
3	Finerenone	47.8	Comparable CV efficacy
4	Placebo	0.8	Background risk
*C. Safety: treatment discontinuation (lower risk = higher rank)*			
1	SGLT2 inhibitor	96.0	Lowest discontinuation; comparable to placebo
2	Placebo	63.2	Reference standard
3	Finerenone	22.8	Higher discontinuation, mainly hyperkalemia
4	Semaglutide	18.0	Higher discontinuation, mainly GI events

SUCRA, values (%) represent the posterior probability of a treatment being ranked among the best; higher values indicate superior ranking.

**FIGURE 3:**
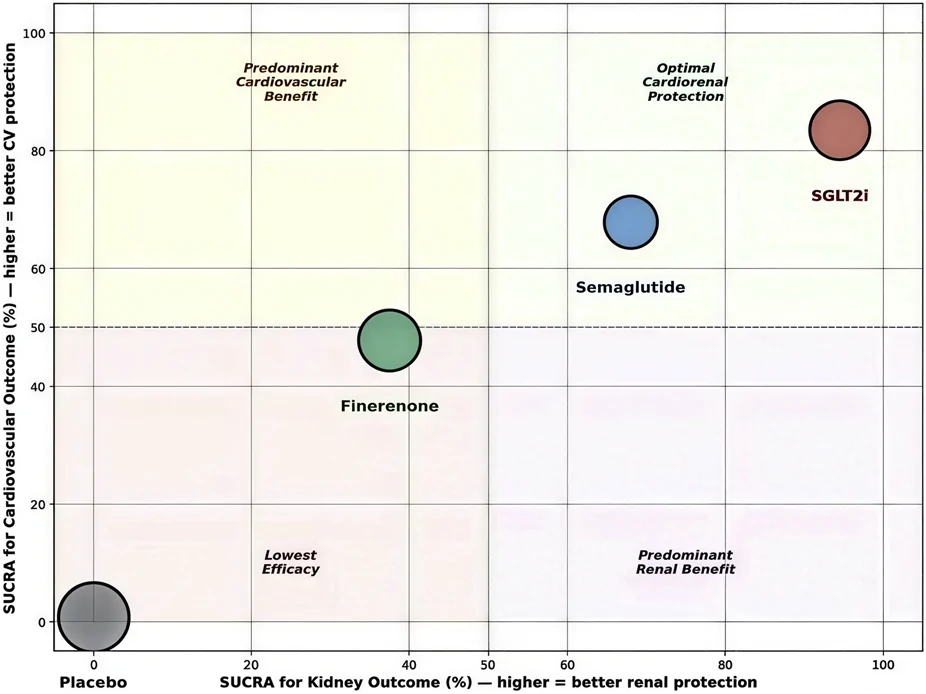
Two-dimensional ranking of interventions based on SUCRA values. SUCRA: Surface Under the Cumulative Ranking Curve (0 = worst ranking, 100 = best ranking). Interventions positioned in the upper-right quadrant indicate optimal combined cardiorenal efficacy.

### 3.8 Sensitivity analyses and robustness

Four pre-specified sensitivity analyses evaluated the robustness of the main findings. Point estimates for hazard ratios were remarkably stable across all four specifications, with median HRs for all three treatments versus placebo differing by no more than 0.01 between specifications. The between-study heterogeneity parameter τ was estimated to be small in all random-effects models (τ = 0.045–0.061), consistent with low true between-trial variation. The WAIC differences between fixed- and random-effects models were minimal.

For the key pairwise comparison of SGLT2 inhibitors versus finerenone on the kidney outcome, the posterior probability of SGLT2i superiority remained ≥96% across all four model specifications (100% under fixed effects with six trials, 96% under random effects with six trials, 100% under fixed effects with seven trials, and 97% under random effects with seven trials). Addition of the SCORED trial did not materially alter the SGLT2 inhibitor pooled estimate or the relative ranking of treatments. Sensitivity E (excluding EMPA-KIDNEY entirely) strengthened rather than attenuated the primary findings: SGLT2i kidney HR 0.68 (95% CrI 0.59–0.77); SGLT2i-vs-finerenone HR 0.80 (95% CrI 0.69–0.94; posterior probability >99.7%), confirming that the EMPA-KIDNEY data approximation does not drive the conclusions.

### 3.9 Model convergence and diagnostics

Markov Chain Monte Carlo sampling achieved excellent convergence in all models (Gelman-Rubin R̂ ≤ 1.01; effective sample sizes >10,000 for all basic parameters). Trace plots and density plots confirmed adequate mixing. Residual deviance was close to the number of data points in all models.

## 4 Discussion

This Bayesian network meta-analysis, incorporating the landmark renal outcome data from the FLOW trial and treating FIDELIO-DKD and FIGARO-DKD as separate evidence nodes, delineates the comparative cardiorenal efficacy and safety of the three contemporary pharmacological therapies evaluated against placebo on a background of renin-angiotensin system (RAS) blockade in patients with type 2 diabetes and chronic kidney disease. Three findings deserve particular emphasis. First, SGLT2 inhibition provides the greatest renal protection (HR 0.70, 95% credible interval 0.64–0.76), with a statistically significant advantage over finerenone in indirect comparison (HR 0.83, 95% CrI 0.73–0.95; posterior probability of superiority = 100%) — a finding that did not emerge from prior network meta-analyses relying on the FIDELITY pooled hazard ratio. Second, semaglutide achieves substantial renal protection (HR 0.76, 95% CrI 0.66–0.88) that is numerically superior to finerenone (HR 0.90, 95% CrI 0.76–1.07; posterior probability of superiority = 88%), though this difference does not reach statistical significance. Third, all three active agents significantly reduce the composite cardiovascular outcome relative to placebo, with no statistically significant differences between active treatments. Together, these findings refine the contemporary framework for diabetic kidney disease pharmacotherapy, which is now most appropriately conceptualized as resting on four pillars: RAS blockade as the long-standing foundation, SGLT2 inhibitors as the essential second pillar, and semaglutide and finerenone as complementary additions guided by individual patient phenotype.

The primacy of SGLT2 inhibitors in our analysis reinforces their established status as the cornerstone add-on therapy for diabetic kidney disease (Kidney Disea se: Improving Global Outcomes KDIGO CKD Work Group, 2024). The mechanistic basis for this primacy—restoration of tubuloglomerular feedback with consequent reduction of intraglomerular hypertension—provides a hemodynamic benefit that is largely independent of glycemic control and operates across a broad spectrum of CKD stages (Cortinovis et al., 2022). Our analysis provides a specific quantitative addition to this evidence: the statistically significant advantage of SGLT2 inhibition over finerenone for kidney protection (HR 0.83, 95% CrI 0.73–0.95) is a finding unique to this network meta-analysis. It did not emerge in earlier analyses that pooled FIDELIO-DKD and FIGARO-DKD via the FIDELITY combined estimate (HR 0.77), in which the comparison against SGLT2 inhibitors yielded credible intervals that crossed unity. Separating the two finerenone trials—as we did to preserve information about their substantively different inclusion criteria, primary endpoints, and follow-up durations—shifted the finerenone pooled kidney hazard ratio to 0.84 and clarified that SGLT2 inhibitors are indirectly superior to finerenone for kidney protection. This methodological refinement illustrates a more general principle: that network meta-analyses are sensitive to decisions about whether to pool statistically related but clinically distinct trials, and that preserving between-trial heterogeneity can produce different and arguably more faithful estimates.

All three drug classes also exert anti-inflammatory effects that may contribute to their renal and cardiovascular protection beyond hemodynamic or metabolic mechanisms. SGLT2 inhibitors attenuate NLRP3 inflammasome activation and reduce circulating interleukin-6 and tumor necrosis factor-α; finerenone directly targets mineralocorticoid receptor-driven inflammation and fibrosis; and semaglutide reduces C-reactive protein and systemic inflammatory markers. These properties are particularly relevant in patients with diabetic CKD, who exhibit chronic low-grade inflammation accelerating both nephropathy progression and cardiovascular events. The heightened susceptibility of diabetic CKD patients to infections—as demonstrated during the COVID-19 pandemic, where diabetes and CKD were independent risk factors for severe morbidity and mortality (Ceriello and Prattichizzo, 2022)—raises the question of whether these anti-inflammatory effects may confer ancillary protection against infection-related complications, although this hypothesis requires dedicated investigation.

The FLOW trial represents a decisive advance in the evidence base for glucagon-like peptide-1 receptor agonists in kidney disease. Prior to FLOW, the renal benefits of this drug class were derived from exploratory secondary analyses of cardiovascular outcome trials (Rossing et al., 2022), a weaker evidentiary foundation that limited widespread nephrology adoption. FLOW established a 24% risk reduction in a composite kidney endpoint as a pre-specified primary outcome (Perkovic et al., 2024), and its incorporation into our network positions semaglutide alongside SGLT2 inhibitors and finerenone as the third evidence-based pharmacological pillar for diabetic kidney disease. Our indirect comparison places semaglutide numerically between SGLT2 inhibitors and finerenone on the kidney ranking (SUCRA 68.0% vs. 94.5% and 37.5%, respectively), with credible intervals that overlap with both. The semaglutide-versus-finerenone comparison (HR 0.90, 95% CrI 0.76–1.07) does not meet formal criteria for statistical superiority, but the posterior probability of semaglutide being the more effective agent for kidney protection is 88%, providing probabilistic support for a hierarchical rather than parity framing. We explicitly acknowledge that our findings apply to semaglutide specifically; extrapolation to the broader GLP-1 receptor agonist class would be premature in the absence of dedicated renal outcome trials with other members of the class, and similar caution applies to treating finerenone as representative of a broader non-steroidal mineralocorticoid receptor antagonist class.

Despite SGLT2 inhibitors’ primacy for renal protection, the choice among add-on therapies in individual patients is appropriately guided by phenotype rather than rank alone. Semaglutide offers distinct advantages for patients with concurrent obesity, established atherosclerotic cardiovascular disease, or suboptimal glycemic control, consistent with the 2024 ADA Standards of Care (American Diabetes Association Professional Practice Committee, 2024). Importantly, the case for semaglutide in patients with atherosclerotic disease is not unique—FIDELIO-DKD and FIGARO-DKD enrolled populations with 45.9% and 45.8% prevalent ASCVD, respectively, and finerenone produced comparable cardiovascular risk reduction (HR 0.87, 95% CrI 0.79–0.95). Finerenone remains particularly well-suited for patients with persistent albuminuria despite SGLT2 inhibitor therapy, given its mechanism targeting mineralocorticoid receptor-driven inflammation and fibrosis, (Barrera-Chimal et al., 2022), (Agarwal et al., 2022) and for patients at elevated heart failure risk, in whom the FIGARO-DKD heart failure benefit was most prominent. The qualitatively different adverse-effect profiles—hyperkalemia with finerenone and gastrointestinal intolerance with semaglutide—also inform choice in ways not captured by an undifferentiated discontinuation outcome, and should be discussed explicitly with patients during shared decision-making.

Beyond the composite cardiovascular endpoints analyzed in our NMA, the broader cardiovascular protection afforded by these agents is particularly relevant given the markedly elevated rates of acute coronary syndromes, revascularization needs, and cardiovascular mortality in patients with diabetic CKD (Cau et al., 2022). SGLT2 inhibitors reduce heart failure hospitalization across trial populations; finerenone demonstrated significant heart failure benefit in FIGARO-DKD; and semaglutide reduced atherosclerotic events in FLOW. Although our NMA found no statistically significant between-agent differences for the composite cardiovascular outcome, the mechanisms of cardiovascular protection differ across classes—hemodynamic unloading for SGLT2 inhibitors, anti-inflammatory and anti-fibrotic effects for finerenone, and anti-atherosclerotic and metabolic benefits for semaglutide—suggesting that combination therapy may provide complementary cardiovascular protection.

Our findings dovetail with, and are substantially strengthened by, the recent publication of the CONFIDENCE trial (Agarwal et al., 2025), which provided the first randomized evidence for combination therapy within this framework. In 818 participants with type 2 diabetes and CKD randomized to finerenone plus empagliflozin, finerenone alone, or empagliflozin alone, combination therapy reduced urinary albumin-to-creatinine ratio by an additional 29% compared with finerenone monotherapy and 32% compared with empagliflozin monotherapy at 180 days, without new safety signals. This provides proof-of-mechanism for additive albuminuria reduction with dual therapy. However, CONFIDENCE was a 6-month surrogate-endpoint trial not powered for hard renal outcomes (ESKD, sustained eGFR decline) or long-term cardiovascular events. Long-term safety signals such as hyperkalemia with dual blockade may emerge over longer follow-up. Our NMA findings are therefore most relevant to informing which monotherapy to initiate first, rather than which combination to prefer. Our indirect-comparison findings complement these combination-therapy results by quantifying the expected monotherapy benefit of each agent, which is necessary for framing the incremental contribution of adding a second or third agent.

The small number of trials per node (three for SGLT2 inhibitors, two for finerenone, one for semaglutide) is the foremost methodological limitation of this analysis. We have addressed this concern in three ways. First, we report pairwise posterior probabilities alongside point estimates and SUCRA rankings, allowing readers to calibrate confidence in each pairwise comparison without overreliance on a single summary metric. Second, we conducted five pre-specified sensitivity analyses—across fixed- and random-effects specifications, with and without the SCORED trial, and excluding EMPA-KIDNEY—and demonstrated that point estimates, SUCRA rankings, and posterior probabilities for all key comparisons are highly robust across specifications (posterior probability of SGLT2 inhibitor superiority to finerenone ≥93% in all five specifications). Third, we restricted our interpretive claims to the specific agents with dedicated primary-endpoint renal trials rather than extrapolating to their broader drug classes.

Several additional limitations warrant attention. The composite kidney outcome was not defined identically across trials: the threshold for sustained eGFR decline was ≥40% in most trials but ≥50% in DAPA-CKD and FLOW; cardiovascular death was a component of the kidney composite in most trials but not in FIDELIO-DKD or FIGARO-DKD. These differences are catalogued in Supplementary Table S2 and could introduce modest heterogeneity that our aggregate-data analysis cannot fully address. We used study-level summary data rather than individual participant data, which precludes granular subgroup analyses by baseline eGFR stratum, albuminuria severity, body mass index, or baseline ASCVD status. Baseline characteristics varied across trials, most notably between FIDELIO-DKD (mean eGFR 44.3) and FIGARO-DKD (mean eGFR 67.8). Follow-up durations ranged from 2.0 to 3.4 years, potentially differentially affecting the detection of late-occurring renal and cardiovascular events. Funnel-plot assessment of publication bias is underpowered with six trials and was not performed; residual publication bias cannot be excluded. The safety outcome (all-cause treatment discontinuation) is a crude composite that obscures the qualitatively distinct adverse-event profiles of the different agents. Finally, and most fundamentally, the star-shaped topology of this network means that all pairwise comparisons between active agents rest on indirect evidence via the shared placebo node and on the plausibility of the transitivity assumption. Only dedicated head-to-head randomized trials can definitively resolve the relative positioning of SGLT2 inhibitors, semaglutide, and finerenone. Although key analytic decisions were documented internally prior to data extraction, the absence of prospective PROSPERO registration increases the risk of unrecognized analytic flexibility. The robustness of results across all five sensitivity analyses provides empirical reassurance, but this limitation should be weighed by readers.

Additionally, all included trials defined diabetic kidney disease using clinical criteria (eGFR and UACR thresholds) rather than kidney biopsy; consequently, the enrolled populations may include patients with non-diabetic kidney pathologies, and differential treatment effects by underlying kidney histology cannot be assessed in this aggregate-data analysis.

Several avenues of future research follow directly from these findings. Dedicated trials evaluating the cardiorenal efficacy and safety of three-way combinations—RAS blockade plus SGLT2 inhibitor plus either semaglutide or finerenone, or the full four-agent combination—are warranted to determine whether the mechanistic complementarity suggested by CONFIDENCE extends to hard renal and cardiovascular endpoints over longer durations. Individual participant data meta-analyses (Riley et al., 2021) incorporating baseline covariates would allow more precise identification of which patient phenotypes derive the greatest benefit from each agent. Head-to-head randomized comparisons between semaglutide and finerenone, and between different SGLT2 inhibitors, would strengthen inference that currently relies on indirect comparison. Finally, dedicated renal outcome trials for additional GLP-1 receptor agonists would clarify whether the renal benefits observed with semaglutide generalize to the drug class (Yi et al., 2026).

## 5 Conclusion

In patients with type 2 diabetes and chronic kidney disease, SGLT2 inhibitors provide the greatest reduction in the risk of kidney failure and disease progression. When the two finerenone trials are analyzed as separate evidence nodes, an indirect statistically significant advantage of SGLT2 inhibitors over finerenone emerges (HR 0.83, 95% CrI 0.73–0.95 [fixed effects]; posterior probability >99.9%). This finding is analytic-choice-dependent, persists across all sensitivity analyses including exclusion of EMPA-KIDNEY, and should be interpreted as hypothesis-generating pending direct randomized evidence. Semaglutide achieves substantial renal protection that is numerically greater than finerenone with a posterior probability of 88%, though without formal statistical significance. All three active agents significantly reduce cardiovascular events relative to placebo, with no statistically significant differences between active treatments. These findings support a four-pillar framework for diabetic kidney disease pharmacotherapy—renin-angiotensin system blockade and SGLT2 inhibition as the foundational combination, with semaglutide and finerenone as complementary, phenotype-matched additions—and provide the probabilistic evidence base for individualized, risk-stratified prescribing in this high-risk population.

## 6 Implications for practice

This Bayesian network meta-analysis, updated with the FLOW trial’s primary renal outcome data and using a more faithful separation of FIDELIO-DKD and FIGARO-DKD, clarifies the relative positioning of contemporary therapies for diabetic kidney disease. SGLT2 inhibitors should be offered to all eligible patients as foundational add-on therapy to renin-angiotensin system blockade, now with indirect evidence showing a significant kidney-protective advantage over finerenone. Following the FLOW trial, semaglutide is established as a robust renal-protective option—with ranking intermediate between SGLT2 inhibitors and finerenone—and is particularly suitable for patients with concurrent obesity, established atherosclerotic cardiovascular disease, or suboptimal glycemic control. Finerenone remains the preferred add-on for patients with persistent albuminuria despite SGLT2 inhibitor therapy or at elevated heart failure risk, and is also supported by cardiovascular benefit in populations with prevalent atherosclerotic disease. The recent CONFIDENCE trial demonstrated that early combination therapy produces additive albuminuria reduction without safety signal, supporting a shift from sequential stepwise prescribing toward earlier individualized combination therapy framed by this four-pillar paradigm.

## Data Availability

The original contributions presented in the study are included in the article/Supplementary Material, further inquiries can be directed to the corresponding authors.
