# Supplementary material for: SGLT2 inhibitors, semaglutide, and finerenone in diabetic kidney disease: a Bayesian network meta-analysis
Source: Front Pharmacol. 2026 Sep 24;17:1882227. doi: 10.3389/fphar.2026.1882227 (PMC13646529; doi:10.3389/fphar.2026.1882227)
Supplement: Supplementary file 1 [file Table1.docx]

**Supplementary Materials**

*Comparative Cardiorenal Efficacy of SGLT2 Inhibitors, Semaglutide, and Finerenone in DKD: A Systematic Review and Bayesian NMA*

## **Contents**

Table S1. Full Database Search Strategy

Table S2. Detailed Outcome Definitions

Table S3. Transitivity Assessment

Table S4. Sensitivity Analysis Results (5 Specifications)

Table S5. All-cause Discontinuation + Sensitivity E

Figure S1. Risk of Bias (RoB 2.0)

Figure S2. Robustness Forest Plots

File 1. Analysis Code (Python/PyMC)

# **Table S1. Full Database Search Strategy**

Searched PubMed, Embase, Cochrane CENTRAL through July 31, 2025. No language or date restrictions.

## **PubMed — 1,734 records**

```
(("Sodium-Glucose Transporter 2 Inhibitors"[Mesh] OR "SGLT2 inhibitor"[tiab]
 OR "dapagliflozin"[tiab] OR "empagliflozin"[tiab] OR "canagliflozin"[tiab] OR "sotagliflozin"[tiab])
 OR ("Glucagon-Like Peptide-1 Receptor"[Mesh] OR "semaglutide"[tiab] OR "liraglutide"[tiab])
 OR ("finerenone"[tiab] OR "non-steroidal mineralocorticoid"[tiab]))
AND (("Diabetes Mellitus, Type 2"[Mesh] OR "type 2 diabetes"[tiab])
 AND ("Renal Insufficiency, Chronic"[Mesh] OR "chronic kidney disease"[tiab]))
AND ("Randomized Controlled Trial"[pt] OR "phase 3"[tiab])
```

## **Embase — 1,512 records**

```
('sodium glucose cotransporter 2 inhibitor'/exp OR dapagliflozin:ti,ab OR empagliflozin:ti,ab
 OR canagliflozin:ti,ab OR semaglutide:ti,ab OR finerenone:ti,ab)
AND ('non insulin dependent diabetes mellitus'/exp)
AND ('chronic kidney disease'/exp) AND ('randomized controlled trial'/exp)
```

## **Cochrane CENTRAL — 206 records**

```
#1 MeSH: [SGLT2 Inhibitors] #2 (dapagliflozin OR empagliflozin OR canagliflozin)
#3 MeSH: [GLP-1 Receptor] #4 (semaglutide) #5 (finerenone)
#6 #1-5 combined; #7 T2D; #8 CKD; #9 #6 AND #7 AND #8
```

## **Combined**

| **Database** | **Records** |
| --- | --- |
| **PubMed** | 1,734 |
| **Embase** | 1,512 |
| **Cochrane** | 206 |
| **Total** | 3,452 |
| **After dedup** | 2,437 |

# **Table S2. Outcome Definitions**

## **Kidney Composite**

| **Trial** | **HR (95% CI)** | **Components** |
| --- | --- | --- |
| **CREDENCE** | 0.70 (0.59–0.82) | ESKD + doubling Scr + renal/CV death |
| **DAPA-CKD (T2D)** | 0.64 (0.52–0.79) | ≥50% eGFR decline + ESKD + renal/CV death |
| **EMPA-KIDNEY** | 0.72 (0.64–0.82) | Kidney progression (≥40% eGFR, ESKD, eGFR<10) + CV death |
| **FIDELIO-DKD** | 0.82 (0.73–0.93) | ESKD + ≥40% eGFR + renal death (no CV death) |
| **FIGARO-DKD** | 0.87 (0.76–1.01) | ESKD + ≥40% eGFR + renal death (secondary endpoint) |
| **FLOW** | 0.76 (0.66–0.88) | Kidney failure (≥50% eGFR, eGFR<15, ESKD) + renal/CV death |

## **CV Composite**

| **Trial** | **HR (95% CI)** | **Components** |
| --- | --- | --- |
| **CREDENCE** | 0.80 (0.67–0.95) | 3-point MACE |
| **DAPA-CKD (T2D)** | 0.70 (0.53–0.92) | CV death + HHF |
| **EMPA-KIDNEY** | 0.84 (0.67–1.07) | CV death + HHF |
| **FIDELIO-DKD** | 0.86 (0.75–0.99) | 4-point MACE |
| **FIGARO-DKD** | 0.87 (0.76–0.98) | 4-point MACE (primary) |
| **FLOW** | 0.82 (0.68–1.00) | 3-point MACE |

*Key heterogeneity: eGFR threshold (40% vs 50%); CV death in kidney composite (absent in FIDELIO/FIGARO); CV composite (3- vs 4-point MACE vs CV death+HHF).*

# **Table S3. Transitivity Assessment**

Effect modifier distributions across placebo arms of included trials.

| **Trial** | **N** | **Age** | **eGFR** | **UACR** | **ASCVD%** | **RAS%** |
| --- | --- | --- | --- | --- | --- | --- |
| **CREDENCE** | 2,199 | 63.0 | 56.2 | 927 | 50.4 | 99.9 |
| **DAPA-CKD** | 1,455 | 64.5 | 43.1 | 1,019 | 45.5 | 98.0 |
| **EMPA-KIDNEY** | 1,541 | 64.2 | 36.9 | 395 | 33.7 | 87.6 |
| **FIDELIO-DKD** | 2,841 | 65.6 | 44.3 | 852 | 45.9 | 99.8 |
| **FIGARO-DKD** | 3,666 | 64.1 | 67.8 | 308 | 45.8 | 99.6 |
| **FLOW** | 1,766 | 66.6 | 47.0 | 568 | 67.5 | 94.5 |

*eGFR mL/min/1.73m²; UACR median mg/g. Range: eGFR 36.9–67.8 (31 units, 2 CKD stages); UACR 308–1,019; ASCVD 33.7–67.5%. Random-effects τ < 0.1 suggests low true effect variation despite baseline differences. Residual transitivity violation cannot be excluded; IPD meta-analysis needed.*

# **Table S4. Sensitivity Analysis Results**

## **A. Kidney HR vs Placebo**

| **Model** | **SGLT2i** | **Semaglutide** | **Finerenone** |
| --- | --- | --- | --- |
| **Main (6, FE)** | 0.70 (0.64–0.76) | 0.76 (0.66–0.88) | 0.84 (0.77–0.92) |
| **Sens A (6, RE)** | 0.70 (0.59–0.81) | 0.76 (0.59–0.99) | 0.84 (0.71–1.03) |
| **Sens B (7+SCORED, FE)** | 0.71 (0.66–0.77) | 0.76 (0.66–0.88) | 0.84 (0.77–0.92) |
| **Sens C (7, RE)** | 0.71 (0.63–0.80) | 0.76 (0.61–0.96) | 0.84 (0.73–0.99) |
| **Sens E (5 excl EMPA, FE)** | 0.68 (0.59–0.77) | 0.76 (0.66–0.88) | 0.84 (0.77–0.92) |

## **B. CV HR vs Placebo**

| **Model** | **SGLT2i** | **Semaglutide** | **Finerenone** |
| --- | --- | --- | --- |
| **Main (6, FE)** | 0.79 (0.70–0.90) | 0.82 (0.68–0.99) | 0.87 (0.79–0.95) |
| **Sens A (6, RE)** | 0.79 (0.65–0.94) | 0.82 (0.59–1.14) | 0.87 (0.71–1.07) |
| **Sens B (7, FE)** | 0.78 (0.71–0.87) | 0.82 (0.68–0.99) | 0.86 (0.79–0.95) |
| **Sens C (7, RE)** | 0.78 (0.68–0.89) | 0.82 (0.64–1.06) | 0.87 (0.74–1.01) |
| **Sens E (5 excl EMPA, FE)** | 0.77 (0.67–0.89) | 0.82 (0.68–0.99) | 0.87 (0.79–0.95) |

## **C. SGLT2i vs Finerenone (Kidney)**

| **Model** | **HR (95% CrI)** | **P(SGLT2i better)** |
| --- | --- | --- |
| **Main (6FE)** | 0.83 (0.73–0.95) | >99.9% |
| **Sens A (6RE)** | 0.83 (0.64–1.04) | 96% |
| **Sens B (7FE)** | 0.85 (0.75–0.96) | >99.9% |
| **Sens C (7RE)** | 0.85 (0.69–1.01) | 97% |
| **Sens E (5 excl EMPA, FE)** | 0.80 (0.69–0.94) | >99.7% |
| **Sens E (5 excl EMPA, RE)** | 0.80 (0.52–1.16) | 92.9% |

## **D. SUCRA Kidney (%)**

| **Treatment** | **Main** | **Sens A** | **Sens B** | **Sens C** | **Sens E** |
| --- | --- | --- | --- | --- | --- |
| **SGLT2i** | 94.5 | 91.1 | 92.4 | 89.7 | 96.1 |
| **Semaglutide** | 68.0 | 66.6 | 69.9 | 69.1 | 66.1 |
| **Finerenone** | 37.5 | 40.2 | 37.7 | 39.9 | 37.7 |
| **Placebo** | 0.0 | 2.1 | 0.0 | 1.4 | 0.1 |

## **E. Heterogeneity τ**

| **τ Model** | **Median** | **95% CrI** |
| --- | --- | --- |
| **Kidney 6T** | 0.054 | 0.002–0.328 |
| **Kidney 7T** | 0.046 | 0.002–0.236 |
| **CV 6T** | 0.061 | 0.002–0.362 |
| **CV 7T** | 0.045 | 0.002–0.236 |
| **Kidney 5T (excl EMPA)** | 0.080 | 0.003–0.531 |

*All τ < 0.1 except Sens E RE (τ=0.08, borderline). Fixed-effects models appropriate as primary.*

# **Table S5. Discontinuation + Sensitivity E**

## **A. All-cause Discontinuation OR**

| **Comparison** | **OR (95% CrI)** | **Note** |
| --- | --- | --- |
| **SGLT2i vs Placebo** | 0.92 (0.82–1.03) | NS |
| **Semaglutide vs Placebo** | 1.12 (0.92–1.37) | NS |
| **Finerenone vs Placebo** | 1.08 (0.99–1.18) | NS |
| **SGLT2i vs Finerenone** | 0.85 (0.73–0.98) | Significant |
| **SGLT2i vs Semaglutide** | 0.82 (0.65–1.02) | NS |
| **Semaglutide vs Finerenone** | 1.04 (0.83–1.29) | NS |

*Heterogeneous composite: GI events (semaglutide), hyperkalemia (finerenone), GU infections (SGLT2i). Cause-specific NMA not feasible.*

## **B. Sensitivity E: Excluding EMPA-KIDNEY (5 Trials, FE)**

| **Outcome** | **SGLT2i** | **Semaglutide** | **Finerenone** |
| --- | --- | --- | --- |
| **Kidney HR** | 0.68 (0.59–0.77) | 0.76 (0.66–0.88) | 0.84 (0.77–0.92) |
| **CV HR** | 0.77 (0.67–0.89) | 0.82 (0.68–0.99) | 0.87 (0.79–0.95) |

| **Pairwise (Kidney)** | **HR** | **P(better)** |
| --- | --- | --- |
| **SGLT2i vs Finerenone** | 0.80 (0.69–0.94) | >99.7% |
| **Sema vs Finerenone** | 0.90 (0.76–1.07) | 87.1% |
| **SGLT2i vs Sema** | 0.89 (0.74–1.07) | 89.0% |

*Exclusion of EMPA-KIDNEY strengthened all findings. SGLT2i kidney HR improved to 0.68; SGLT2i-vs-finerenone advantage more pronounced (HR 0.80, P >99.7%).*

# **Figure S1. Risk of Bias (RoB 2.0)**

*All six trials: low risk across all five RoB 2.0 domains.*

# **Figure S2. Robustness Forest Plots**

## **A. Kidney Outcomes**

*Point estimates stable (ΔHR ≤0.01). CrIs widen under RE but direction preserved.*

## **B. CV Outcomes**

## **C. SUCRA Stability**

*Ranking SGLT2i > Semaglutide > Finerenone preserved in all specifications.*

# **File 1. Analysis Code**

Python/PyMC code for the Bayesian NMA. Full files: run_nma_main.py and run_sensitivity.py.

```
import numpy as np; import pymc as pm

kidney_data = {
  'CREDENCE':     {'treat':1, 'hr':0.70, 'lo':0.59, 'hi':0.82},
  'DAPA-CKD_T2D': {'treat':1, 'hr':0.64, 'lo':0.52, 'hi':0.79},
  'EMPA-KIDNEY':  {'treat':1, 'hr':0.72, 'lo':0.64, 'hi':0.82},
  'FIDELIO-DKD':  {'treat':3, 'hr':0.82, 'lo':0.73, 'hi':0.93},
  'FIGARO-DKD':   {'treat':3, 'hr':0.87, 'lo':0.76, 'hi':1.01},
  'FLOW':         {'treat':2, 'hr':0.76, 'lo':0.66, 'hi':0.88},
}
# SE = (log(hi)-log(lo))/(2*1.96)
# Fixed: d_k ~ Normal(0,9); y ~ Normal(d[t], se)
# Random: + tau ~ HalfNormal(0,0.5); theta = d[t] + raw*tau
# MCMC: 4 chains x 5000 draws, 2000 tune, NUTS
# SUCRA: rank posterior, P(rank=k), sum(cum)/K-1
```

Complete .py files available from corresponding author upon request.
